# Coacervate Vesicles as Adaptive Platforms for Synthetic Biology and Smart Materials

**DOI:** 10.1002/chem.202502268

**Published:** 2025-08-19

**Authors:** Francesco Vicentini, Federica Battistin, Pierangelo Gobbo

**Affiliations:** ^1^ Department of Chemical and Pharmaceutical Sciences University of Trieste Via L. Giorgieri 1 Trieste 34127 Italy; ^2^ National Interuniversity Consortium of Materials Science and Technology Unit of Trieste Via G. Giusti 9 Firenze 50121 Italy

**Keywords:** bottom‐up synthetic biology, coacervate, out‐of‐equilibrium, protocell, vesicle

## Abstract

Coacervate vesicles represent a versatile and emerging class of protocells that combine the dynamic properties of coacervate microdroplets with the structural advantages of membrane‐bound systems. Leveraging features such as selective molecular uptake, enhanced reactivity, and dynamicity, they offer a promising platform for both fundamental research and technological applications in synthetic biology. In this concept, we introduce a classification of coacervate vesicles based on their formation mechanisms and energetic landscapes, highlighting how their formation routes give rise to protocells with distinct structural and dynamic properties. Furthermore, we explore how these features translate into potential applications, including artificial life‐like systems, complex communication networks, adaptive soft materials, and smart drug‐delivery platforms. While the field is still in its infancy, the simplicity, versatility, and programmability of coacervate vesicles position them as a powerful framework for engineering next‐generation synthetic life‐like systems.

## Graphical abstract

Coacervate vesicles are emerging protocells that merge coacervates’ functionality within membranised architectures. Their diverse formation mechanisms from coacervate droplets yield tunable, responsive systems with broad potential, from synthetic biology to smart materials, offering a powerful, programmable platform for next‐generation bioinspired technologies.

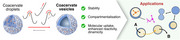

## 1 Introduction

Bottom‐up synthetic biology aims to construct synthetic cells, or protocells, leveraging diverse molecular systems and materials to create cell‐like architectures unconstrained by biological evolution.^[^ ^1^, ^2^ ^]^ While most protocells rely on a self‐assembled membrane for compartmentalisation, which is a central feature in engineering life‐like behaviours,^[^ ^3^, ^4^ ^]^ complex coacervate microdroplets offer a compelling membraneless alternative. They form via associative liquid–liquid phase separation of polyelectrolytes in dilute aqueous solutions, typically driven by weak electrostatic interactions, and can be assembled from a wide range of components, including polymers, biomolecules, and surfactants.^[^ ^5^ ^]^ Their condensed internal phase enables them to concentrate and protect reactants (such as small molecules, nucleic acids, and proteins),^[^ ^6^, ^7^ ^]^ support, and facilitate enzymatic reactions,^[^ ^8^ ^]^ and dynamically evolve.^[^ ^9^, ^10^ ^]^

**Figure 1:**
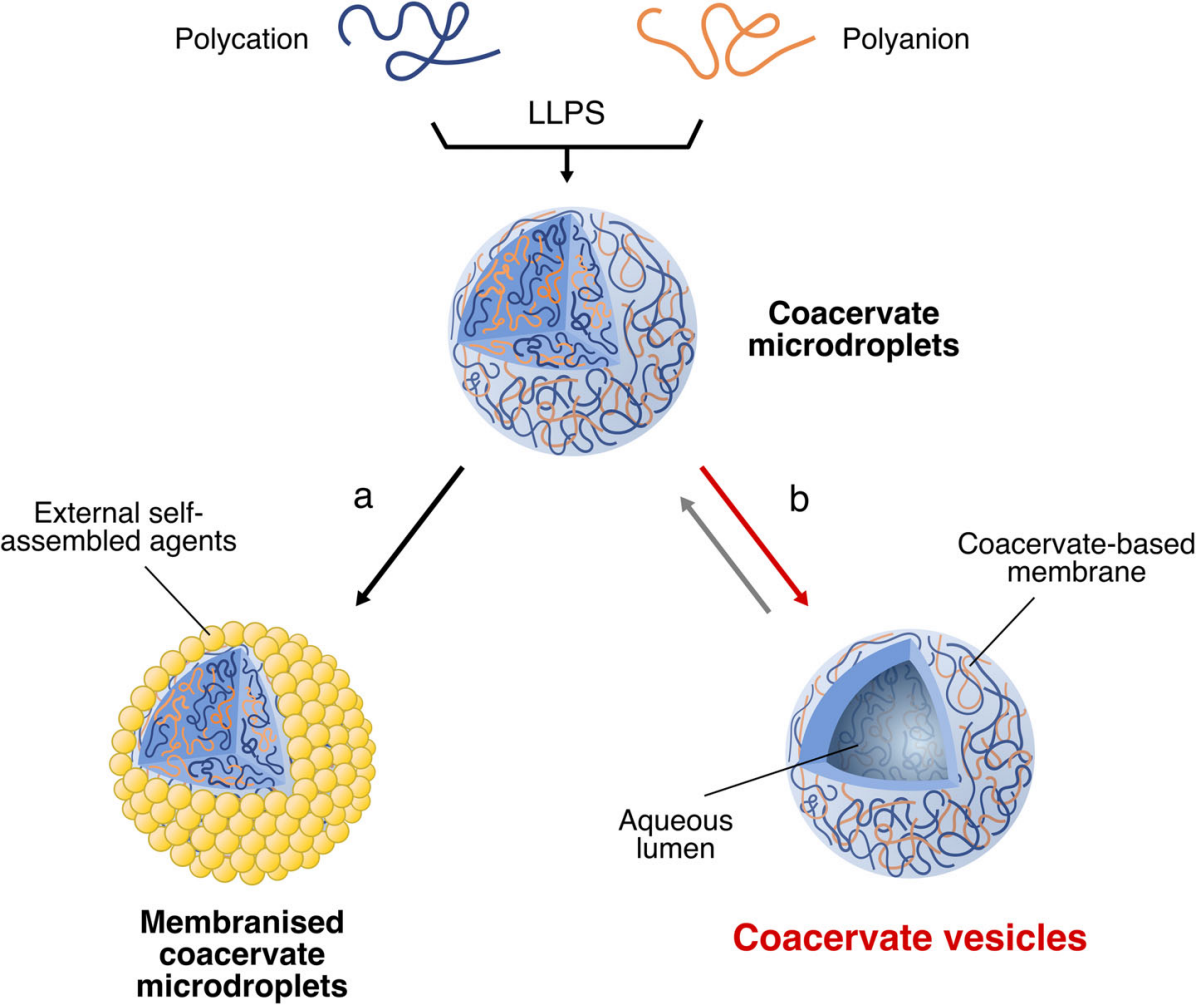
Schematic illustration of the two pathways to stabilise complex coacervate microdroplets. Initially, two oppositely charged polyelectrolytes in solution (polycation in blue and a polyanion in orange) undergo liquid–liquid phase separation (LLPS) to form coacervate microdroplets. These droplets can then be stabilised through two main pathways: **a)** irreversible membranisation with external chemical agents (yellow spheres), **b)** reversible droplet‐to‐vesicle reconfiguration to form hollow coacervate vesicles. Throughout this article, polycations are represented in blue and polyanions in orange for visual consistency.

While promising, coacervate microdroplets suffer from low interfacial tension and weak cohesion, which drive destabilizing coalescence and surface wetting,^[^ ^11^ ^]^ limiting their technological applicability. To address these limitations and harness the beneficial features of coacervates, recent research has shifted toward developing membrane‐bound coacervates.^[^ ^12^ ^]^ To achieve this, two main strategies have emerged: (a) employing external chemical agents that self‐assemble at the water‐droplet interface to stabilize the system,^[^ ^13^ ^]^ or, more intriguingly, (b) triggering structural reconfiguration to generate robust coacervate vesicles (Figure 1). While the first strategy is now well established,^[^ ^14^ ^]^ the formation of coacervate vesicles remains relatively underexplored. These latter systems represent a unique hybrid architecture, combining the molecular richness and responsiveness of coacervates with the structural stability and compartmentalisation of vesicles, alongside reversible chemical processes. This synergy positions coacervate vesicles as an exceptionally versatile and minimalist protocell model.

In this concept, we highlight coacervate vesicles as a young and rapidly evolving protocell model. We classify coacervate vesicles based on their formation mechanisms, review current synthetic strategies, and outline key design principles. We then examine their emerging applications in synthetic biology and biomedicine, highlighting opportunities for next‐generation bioinspired technologies. As dynamic, programmable materials, coacervate vesicles hold great promise for future breakthroughs, ranging from artificial tissues to smart therapeutics, positioning them as powerful platforms at the interface of chemistry, biology, and materials science.

## 2 Formation of Coacervate Vesicles

In this section, we classify coacervate vesicles based on their formation pathway, beginning from complex coacervate microdroplets, into three groups: irreversible, reversible, and nonequilibrium. *Irreversible coacervate vesicles* form through permanent structural changes, typically via strong morphogenic agents or irreversible chemical modifications, resulting in exceptional stability. *Reversible coacervate vesicles* maintain dynamic interconvertibility with their coacervate droplet precursors, enabling switchable functionalities. Lastly, *non‐equilibrium coacervate vesicles* represent the most intriguing class, sustained by a continuous input of energy that maintains the vesicular architecture in a far‐from‐equilibrium state. Upon cessation of energy supply, the system reverts to its original thermodynamic equilibrium. This characteristic renders them particularly relevant as minimal, active (or “living”) model microcompartments.

This classification is critical because their physicochemical properties and thus potential applications are inherently tied to their formation mechanisms. Understanding these distinct pathways will guide future advances in the rational design of functional coacervate vesicles for synthetic biology and bioinspired materials.

### 2.1 Irreversible Coacervate Vesicles

**Figure 2:**
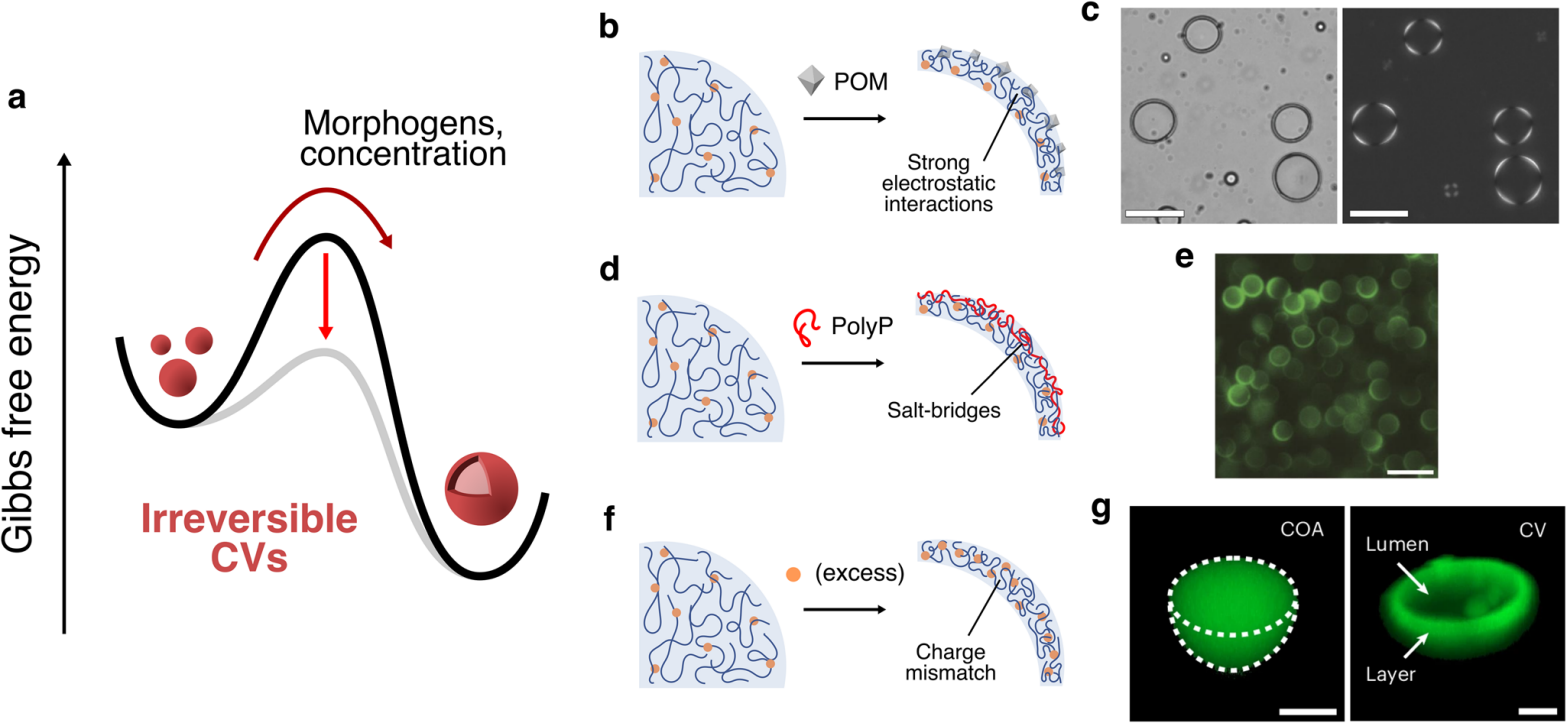
Irreversible formation of coacervate vesicles. **a)** Schematic free energy diagram illustrating the irreversible formation pathway of coacervate vesicles. The addition of a morphogenic agent or changes in polyelectrolyte concentration can trigger a one‐way structural reconfiguration from coacervate microdroplets into more thermodynamically stable coacervate vesicles. **b)** Schematic illustration of the formation of POM‐stabilised PDDA/ATP coacervate vesicles, initiated by strong electrostatic interactions between POM and PDDA, followed by vacuolisation driven by osmotic pressure. In all the schemes, polycations are represented in blue and polyanions in orange. **c)** Brightfield and cross‐polarised microscopy images of micrometre‐sized PDDA/ATP/POM coacervate vesicles. The Maltese‐cross pattern visible with polarised light microscopy is indicative of birefringence of the vesicular membrane. Scale bars = 50 µm. Reprinted with permission from ref. [15]. Copyright 2014 John Wiley and Sons. **d)** Schematic illustration of the formation of polyarginine/ATP coacervate vesicles driven by salt‐bridge interactions between negatively charged polyphosphates (polyP, red line) and the polycation inside the droplet. **e)** Confocal microscopy image of coacervate vesicles taken 1 minute after polyP injection. Scale bar = 10 µm. Green fluorescence is due to the use of a tagged ATP analogue. Reprinted with permission from ref. [19]. Copyright 2025 American Chemical Society. **f)** Schematic illustration of the preparation of nucleoprotein/DNA coacervate vesicles driven by high polyanion concentration, with subsequent charge unbalance and exposure of hydrophobic regions in the polycation. **g)** Structural comparison of coacervate microdroplets (COA, left) and coacervate vesicles (CV, right) via 3D fluorescence microscopy. Scale bars = 1 µm. Green fluorescence is due to fluorescein‐tagged polyanion. Reprinted with permission from ref. [20] Copyright 2025 Springer Nature.

To address coacervate droplet instability, researchers have developed irreversible conversion strategies that yield thermodynamically stable vesicles. This transition prevents reversion to droplets under normal conditions (Figure 2a).

Among these strategies, one of the most widely adopted approaches involves the addition of densely charged species, called “morphogenic agent”, that interact selectively with one of the coacervate components, inducing a spatial reorganisation that ultimately results in vesicle formation. This mechanism was first demonstrated by Williams et al., who discovered that phosphotungstic acid, an inorganic polyoxometalate (POM), can strongly bind to the positively charged polymer poly(diallyldimethylammonium chloride) (PDDA), transforming PDDA/ATP coacervate microdroplets into membrane‐bound vesicles based on osmotic pressure (Figure 2b,c).^[^ ^15^ ^]^ The resulting POM‐coacervate vesicles exhibit a tightly packed, negatively charged membrane encapsulating a PDDA/ATP coacervate sub‐shell. Remarkably, the membranes display pronounced birefringence, suggesting a radially organised internal structure.^[^ ^16^ ^]^ This strategy has since been generalised: by employing mixtures of different POMs, it became possible to introduce catalytic functionalities within the vesicle structure,^[^ ^17^ ^]^ while alternative morphogenic agents, such as sodium dodecyl sulfate, have been used to demonstrate the broader applicability of the vacuolisation process.^[^ ^18^ ^]^ Similarly, recent work has shown that negatively charged polyphosphates can also be used as a morphogenic agent and trigger the reconfiguration of biomolecular complex coacervates into stable coacervate vesicles by forming strong salt‐bridges with the positive component of the droplets (Figure 2d,e).^[^ ^19^ ^]^

Beyond the use of external morphogens, an excess of one of the components of a coacervate microdroplet can similarly form vesicles through charge disproportion, driven by hydrophobic and attractive electrostatic interactions between the two polyelectrolytes. This imbalance modifies and strengthens internal electrostatic forces, spontaneously driving vesicle formation without the need for external additives, thanks to osmotic pressure differences. (Figure 2f,g).^[^ ^20^, ^21^ ^]^

### 2.2 Reversible Coacervate Vesicles

**Figure 3:**
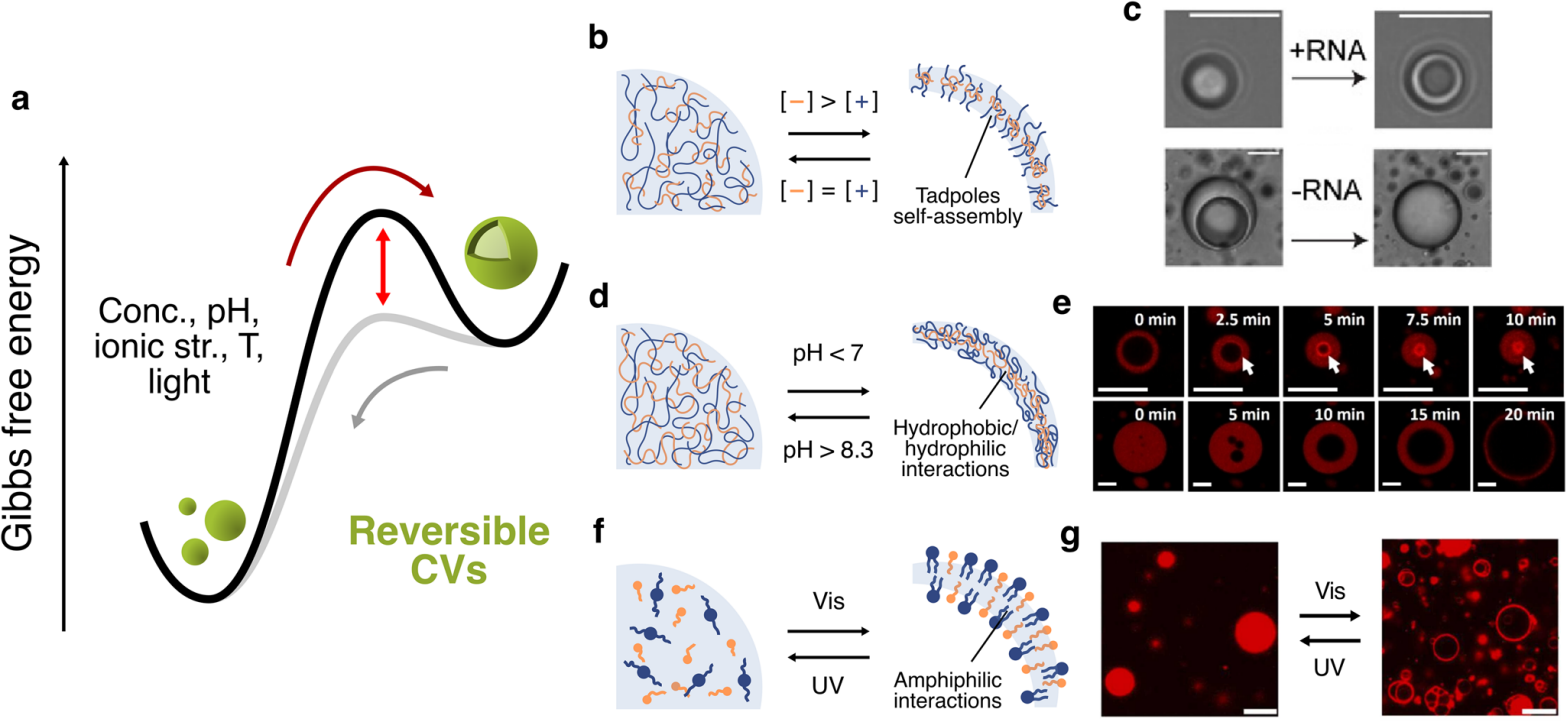
Reversible formation of coacervate vesicles. **a)** Schematic free energy diagram illustrating the reversible formation of coacervate vesicles. These structures can be generated through various strategies that exploit the dynamic and stimuli‐responsive nature of coacervates (red arrow). The transition back to coacervate droplets can be achieved by reversing the applied stimulus (grey arrow). **b)** Schematic of reversible RNA/peptide coacervate vesicles, formed by the self‐assembly of charged tadpole‐like complexes that emerge under RNA‐excess conditions. In all the schemes, polycations are represented in blue and polyanions in orange. **c)** Brightfield microscopy images show how an excess of RNA can drive the reversible formation of coacervate vesicles from RNA/peptide complex droplets.^[^ ^23^ ^]^ **d)** Schematic of the reversible formation of silk fibroin/alginate coacervate vesicles driven by the emergence of hydrophobic patches in the fibroin at certain pH values, forming a three‐tiered membrane. **e)** Time‐resolved microscopy images of an enzyme‐containing coacervate vesicle and coacervate microdroplet that undergo structural transitions into droplets or vesicles, respectively, upon addition of specific enzyme substrates (urea/glucose). Scale bars = 10 µm. Red fluorescence is referred to rhodamine‐tagged silk fibroin.^[^ ^26^ ^]^ **f)** Schematic of the reconfiguration of azobenzene‐containing cationic amphiphiles/sodium dodecyl sulfate coacervate microdroplets into lipid vesicles due to light irradiation. **g)** Confocal microscopy image of the light‐controlled reversible transition between coacervate microdroplets and lipid vesicles using photoresponsive azobenzene cationic surfactants, which isomerise from linear to bent form. UV light induces droplet formation, while visible light restores the vesicles. Scale bars = 20 µm. Red fluorescence is due to sequestered Nile red. Adapted with permission from ref. [34] Copyright 2023 Elsevier.

While irreversible vesicle formation enhances structural stability, it comes at the expense of the intrinsic dynamism characteristic of coacervate systems. To overcome this, researchers have exploited the intrinsic chemical responsiveness of coacervate components to design reversible coacervate vesicles that can switch between two equilibrium states. In these systems, the vesicle configuration represents a relative free energy minimum, accessible by lowering the energy barrier via external stimuli, while the system spontaneously reverts to the more stable droplet state upon stimulus inversion (Figure 3a).

In this context, the ratio between the charged components that initially form the droplets can influence the reconfiguration into vesicles.^[^ ^22^ ^]^ For instance, an excess of RNA in RNA/peptide coacervates leads to stable, birefringent hollow vesicles. These can then revert to droplets once the balance between the two components is restored when peptide solution is added (Figure 3b,c).^[^ ^23^ ^]^ The proposed formation mechanism involves the self‐assembly of surfactant‐like complexes, with a complex coacervate head and a charged tail, that form in far‐from‐equal polyanion/polycation stoichiometry.^[^ ^24^ ^]^ Remarkably, this behaviour extends to other binary polyelectrolyte systems, such as poly(allylamine)‐inorganic polyphosphate.

Beyond stoichiometry, stimuli‐responsive polyelectrolytes offer versatile control over vesicle dynamics. Variations in pH and ionic strength modulate electrostatic interactions within coacervates, enabling reversible transitions to vesicular architectures.^[^ ^25^, ^26^ ^]^ It has also been shown how these environmental cues can be sensed and transduced internally. For example, enzyme‐containing silk fibroin/alginate coacervate microdroplets have been shown to dynamically reorganize into vesicles by electrostatic modifications of the charged fibroin, driven by pH changes caused by enzymatic activity, creating autonomous, signal‐responsive systems (Figure 3d,e).^[^ ^27^, ^28^ ^]^

Temperature can also play a role, particularly in droplets composed of globular proteins, where heating triggers reversible internal rearrangements of protein sites. These internal changes create amphiphilic complexes that self‐assemble to form coacervate vesicles.^[^ ^29^, ^30^ ^]^

Coacervate systems composed of fatty acids and oppositely charged macromolecules can also reversibly form vesicular structures in response to specific stimuli.^[^ ^13^ ^]^ In these terms, concentration,^[^ ^31^ ^]^ pH,^[^ ^32^ ^]^ temperature,^[^ ^33^ ^]^ reversible chemical reactions,^[^ ^34^ ^]^ and light (Figure 3f,g)^[^ ^35^ ^]^ have been used to trigger vesicle formation. However, once formed, these vesicles typically lose the defining characteristics of coacervates, such as dynamic molecular uptake and internal fluidity, and instead resemble traditional lipid vesicles. As such, despite their shared coacervate origin, these lipid structures fall outside the strict definition of coacervate vesicles.

### 2.3 Non‐Equilibrium Coacervate Vesicles

**Figure 4:**
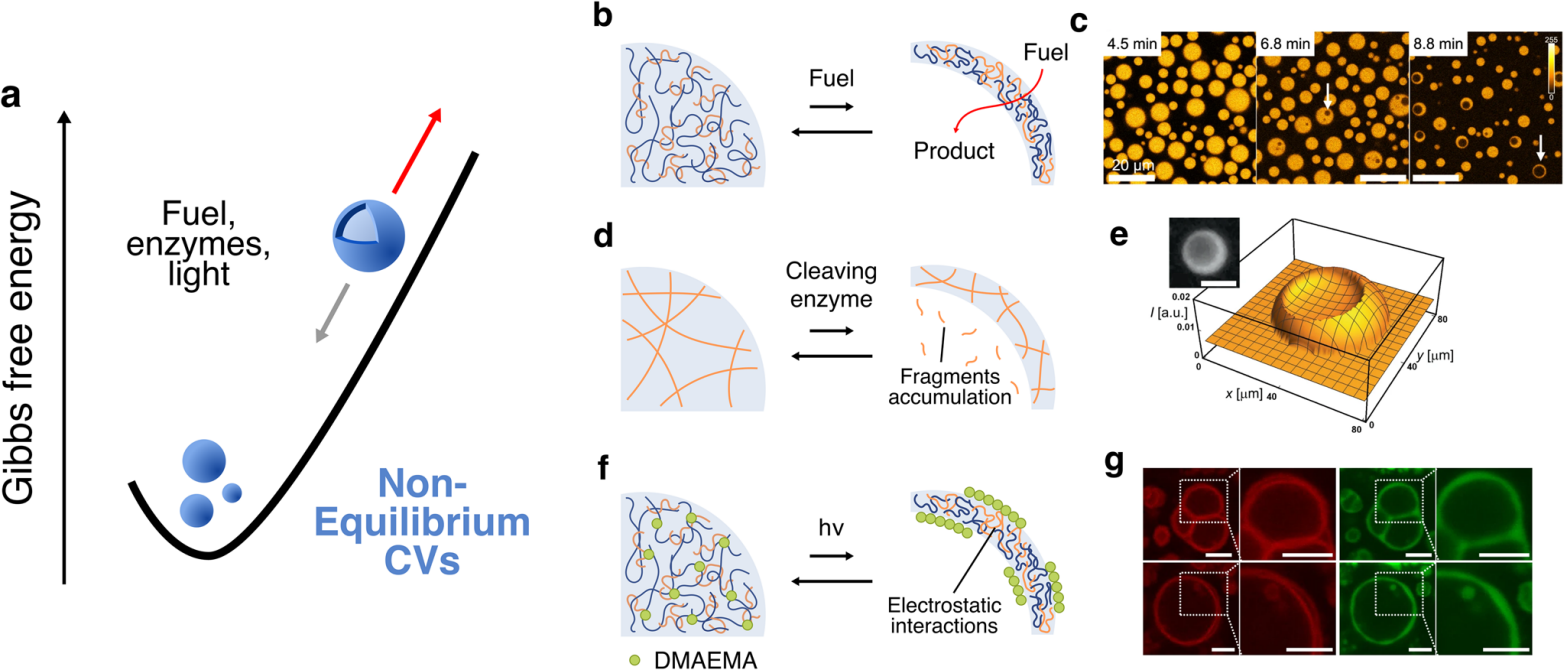
Non‐equilibrium formation of coacervate vesicles. **a)** Schematic free energy diagram illustrating the dissipative self‐assembly of coacervate vesicles. The vesicular structure dissipates once the energy input is removed. **b)** Schematic of non‐equilibrium active peptide/polystyrene sulfonate coacervate vesicles formation: fuel consumption and reaction product accumulation within the droplets create osmotic gradients that drive droplet vacuolisation. In all the schemes, polycations are represented in blue and polyanions in orange. **c)** Time‐resolved confocal microscopy images showing fuel‐driven transition of active coacervate droplets into coacervate vesicles. Scale bar = 20 µm. Yellow fluorescence is referred to sulforhodamine B, sequestered inside the coacervate phase.^[^ ^37^ ^]^ **d)** Schematic of DNA‐based coacervate vesicle formation, driven by enzymatic cleavage and the accumulation of DNA fragments, which induce internal lumen formation and droplet reconfiguration. **e)** 3D microscopy reconstruction of a DNA‐based droplet undergoing vacuole formation due to the accumulation of DNA fragments. Scale bar = 40 µm.^[^ ^38^ ^]^ **f)** Schematic of the formation of coacervate vesicles via photoinduced polymerisation of dimethylaminoethyl methacrylate (DMAEMA, green circles). The resulting polymer accumulates inside and outside the PDDA/hyaluronic acid coacervate droplet, generating an osmotic pressure gradient that drives water influx and vacuolisation. **g)** Confocal microscopy images showing the formation of multi‐ and single‐compartmentalised coacervate vesicles via visible‐light‐induced polymerisation within PDDA‐hyaluronic acid coacervates. Scale bars = 3 µm. Red and green fluorescence are related to rhodamine B and calcein, respectively. Reprinted with permission from ref. [40]. Copyright 2025 Springer Nature.

In contrast to equilibrium systems, where vesicle formation reflects stable or reversible thermodynamic states, placed in relative *minima* of Gibbs free energy, non‐equilibrium coacervate vesicles require continuous energy consumption to maintain their structural reconfiguration (Figure 4a). These systems are inherently dynamic, often transient, and represent a conceptual leap toward mimicking the dissipative self‐assembly behaviours seen in living cells and tissues.^[^ ^36^, ^37^ ^]^

Most non‐equilibrium coacervate vesicles are driven by fuel‐consuming chemical reactions, in which the transformation of high‐energy precursors spatially reorganizes the system over time. For example, it has been elegantly demonstrated that active coacervate droplets, which respond to external chemical fuels, can convert into spherical vesicles upon the consumption of a carbodiimide‐based chemical fuel. This fuel transiently modifies the polycation component of the coacervate, shifting the balance of interactions and generating an osmotic gradient that leads to vesicle formation (Figure 4b,c).^[^ ^38^ ^]^ Upon fuel depletion, the reverse hydrolysis reaction restores the original molecular state, the osmotic gradient dissipates, and the vesicle collapses. Similarly, DNA nanostar‐based coacervate microdroplets form dissipative vesicles when subjected to cleaving enzymes. These enzymes, sequestered inside the droplets, break the DNA into shorter fragments that accumulate internally. This buildup of smaller, charged molecules creates internal pressure and structural asymmetry, driving vesicle formation, which is sustained only while the enzymatic cycle persists (Figure 4d,e).^[^ ^39^ ^]^

Osmotic effects also govern light‐fuelled non‐equilibrium systems, where the photoinduced polymerisation of dimethyl aminoethyl methacrylate acts as a transient energy source. Upon visible light exposure, the growing polymer chains accumulate inside and outside PDDA/hyaluronic acid coacervate droplets, increasing the internal charge density. This imbalance drives osmotic water influx, leading to internal vacuole formation and reorganisation into vesicle‐like architectures (Figure 4f,g).^[^ ^40^ ^]^ Though these coacervate vesicles exhibit exceptional longevity (up to 300 days in ambient conditions), they remain dissipative, eventually returning to dense coacervate droplets once light stimulation ceases. A similar dissipative disassembly mechanism was also observed in flow‐driven systems, where coacervate vesicles formed under constant polyelectrolyte influx, and then gradually reverted to coacervate microdroplets when the input stopped.^[^ ^41^ ^]^

## 3 Functional Diversity and Emerging Applications

**Figure 5:**
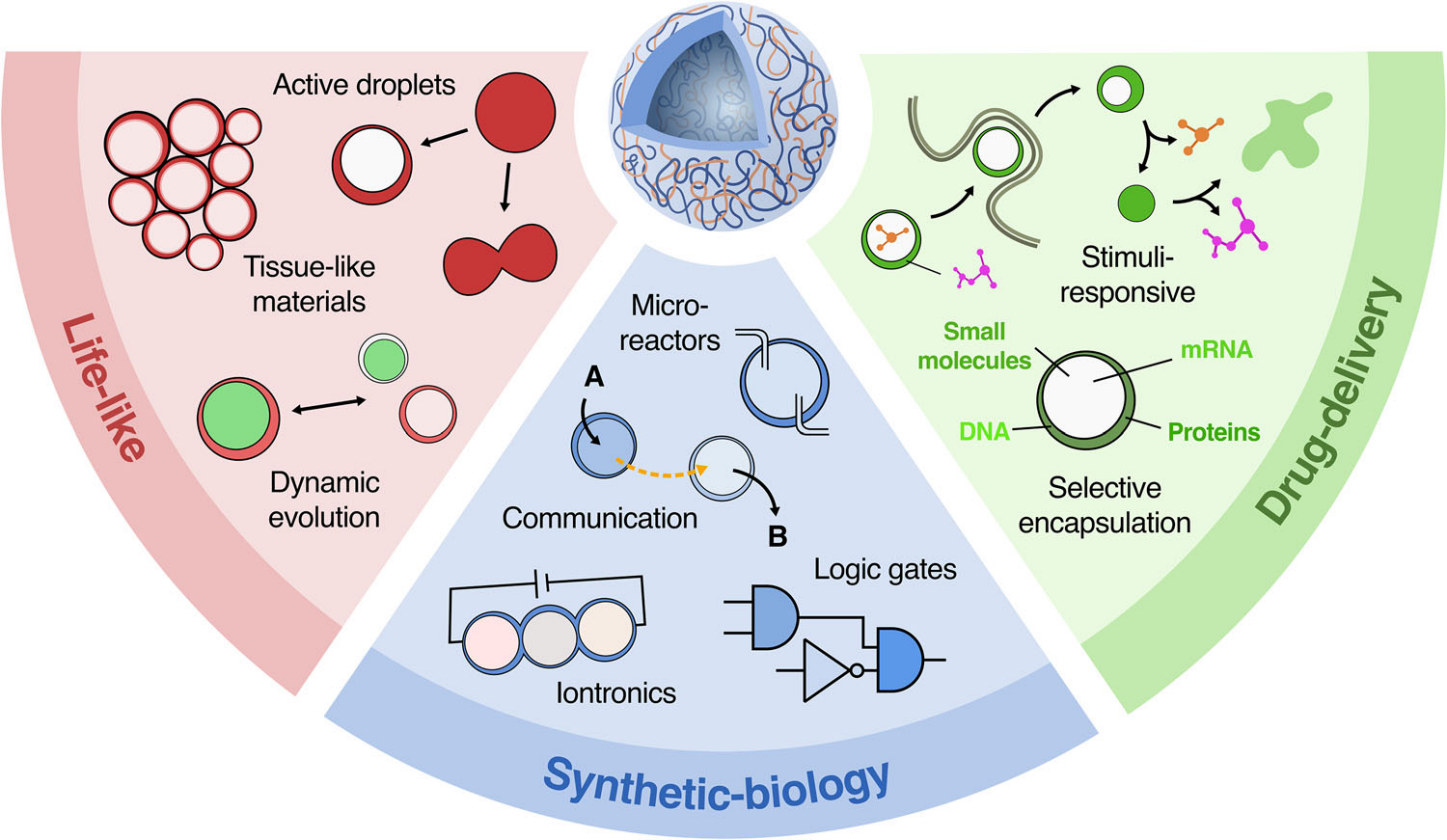
Schematic overview of current and potential future applications of coacervate vesicles.

The physicochemical characteristics of coacervate vesicles, directly dictated by how they are formed, underpin their functional behaviours. This property‐function relationship not only clarifies current applications, but also reveals a broad and still unfolding landscape of future opportunities (Figure 5).

### 3.1 Life‐Like Protocells

In the 1920s, Oparin proposed coacervates as plausible precursors to modern cells,^[^ ^42^, ^43^ ^]^ connecting physical and biological sciences. However, their membraneless nature leaves a plot gap in explaining the transition to modern membrane‐bound cells.

**Figure 6:**
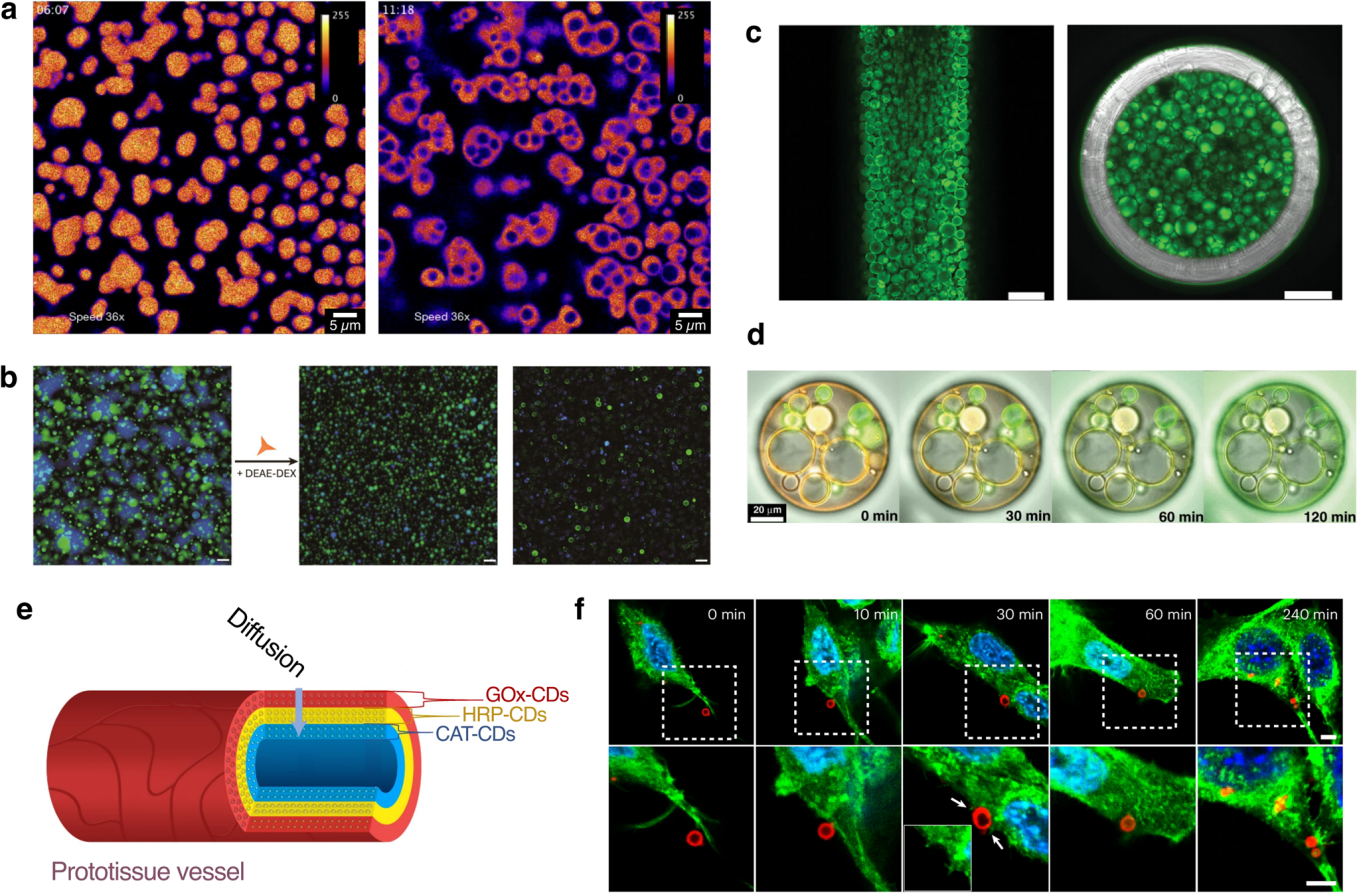
Various examples of emerging applications for coacervate vesicles. **a)** Time‐dependent confocal microscopy images of active peptide/polystyrene sulfonate coacervate droplets showing growth, lumen formation, division, and disassembly. Fluorescence is due to cyanine5, used to label the polyanion.^[^ ^45^ ^]^ **b)** Confocal microscopy images before and after the addition of diethylaminoethyl dextran to a mixed population of two distinct types of polyarginine/ATP and NADPH coacervate vesicles, forming a binary tissue‐like material. Scale bars = 10 µm. Green and blue fluorescence are referred to fluorescently labelled ATP and naturally fluorescent NADPH, respectively. Reprinted with permission from ref. [19]. Copyright 2025 American Chemical Society. **c)** Confocal microscopy images of a glass column reactor densely packed with fluorescein‐tagged enzyme‐containing POM‐coacervate vesicles. Scale bars = 200 µm.^[^ ^51^ ^]^ **d)** Time‐lapse merged fluorescence and brightfield microscopy images of a mixed population of communicating POM‐coacervate vesicles confined in a water‐in‐oil droplet. The appearance of the fluorescent product is observable over time as the result of two competing processes.^[^ ^17^ ^]^ **e)** Schematic of a three‐tiered tissue‐like material with diffusive communication between enzyme‐loaded membranised coacervate droplets.^[^ ^53^ ^]^ **f)** Confocal microscopy images of the different endocytosis stages of a nucleoprotein/DNA CV. The cell skeleton and nucleus were tagged green and blue, respectively. Scale bars = 2 µm. Adapted with permission from ref. [20]. Copyright 2025 Springer Nature.

In this context, non‐equilibrium coacervate vesicles better mimic the dissipative nature of living systems, representing a promising step toward life‐like behaviour. Active coacervate microdroplets have been shown to grow and divide,^[^ ^44^, ^45^ ^]^ and it is not implausible to envision chemical processes within such dynamic droplets giving rise to aqueous lumens and membrane‐like shells (Figure 6a), forming primitive membranised structures.^[^ ^38^, ^46^ ^]^

By tuning the coacervate vesicles’ composition, different (bio)molecules can be selectively partitioned into the coacervate shell or aqueous core, enabling spatially controlled chemical reactivity.^[^ ^23^ ^]^ Reversible coacervate vesicles also support cycles of disassembly and reassembly, allowing them to acquire and retain new functionalities over time, an essential feature of evolutionary systems.^[^ ^30^ ^]^

Finally, the chemical identity of the vesicle membrane can be exploited for higher‐order assembly and communication. Irreversible coacervate vesicles have already been shown to form tissue‐like aggregates through salt‐bridge interactions between diethylaminoethyl dextran and polypeptides in the coacervate vesicle membrane (Figure 6b),^[^ ^19^ ^]^ or between extracellular PDDA and catalytic POM‐coacervate vesicles.^[^ ^47^ ^]^ Looking forward, this strategy could be expanded to include supramolecular or covalent crosslinking approaches.^[^ ^48^, ^49^ ^]^

### 3.2 Synthetic Biology

Capitalizing on the coacervate's inherent capabilities, such as accelerating chemical reactions and selective molecular sequestration,^[^ ^50^, ^51^ ^]^ coacervate vesicles can be envisioned as micrometre‐scale chemical reactors, offering enhanced stability, modularity, and tunability. For instance, enzymes have been selectively partitioned into the coacervate shell of POM‐coacervate vesicles, which can then be integrated into flow reactors to support multi‐enzyme catalysis and mimic key steps of metabolic pathways (Figure 6c).^[^ ^52^ ^]^ Remarkably, similar POM‐coacervate vesicles assembled from inorganic Ru_4_POM catalysts can carry out catalase‐like reactions in fully nonliving systems,^[^ ^17^ ^]^ and can be further used in more complex systems for photocatalytic water oxidation to generate oxygen.^[^ ^47^ ^]^ Despite these advances, the integration of broader enzyme classes or artificial syzymes remains largely unexplored.^[^ ^8^, ^53^ ^]^

Building on this biochemical versatility, coacervate vesicles can be engineered for more advanced tasks, such as protocell–protocell communication, where their responsiveness and compartmentalisation offer a powerful platform. POM‐based irreversible coacervate vesicles have been integrated into competitive protocell networks that respond collectively to input stimuli (Figure 6d).^[^ ^17^ ^]^ Liu et al. extended this concept by embedding enzyme‐loaded coacervate microdroplets in a gel matrix to build tubular tissues capable of decoding external cues and generating coordinated, visible outputs (Figure 6e).^[^ ^54^ ^]^ Future systems could incorporate this functionality within standalone coacervate vesicles, eliminating the need for external scaffolds. Moreover, the intrinsic responsiveness of reversible and non‐equilibrium coacervate vesicles to stimuli, such as changes in light, pH, or ionic strength, can add additional layers of programmability. This could enable coacervate vesicles to act as chemical logic units within distributed computing systems.^[^ ^55^ ^]^

### 3.3 Drug‐Delivery

Coacervate vesicles are emerging as promising platforms for drug delivery, offering a distinct set of advantages over conventional vesicular carriers. Their biphasic structure allows for the simultaneous encapsulation of hydrophilic components in the internal aqueous lumen and charged or hydrophobic ones in the coacervate membrane, enabling tailored multidrug delivery.^[^ ^56^ ^]^ Their intrinsic responsiveness to environmental cues can be harnessed for controlled or site‐specific release.^[^ ^57^ ^]^ For instance, Wen et al. recently reported a stable system based on biocompatible nucleoprotein/DNA scaffolds, capable of selectively trapping oncolytic viruses in the coacervate vesicle membrane and releasing them inside target cells (Figure 6f).^[^ ^20^ ^]^

Moreover, their spontaneous formation under mild, fully aqueous conditions stands out in a field often dominated by complex, over‐engineered carriers designed for fragile therapeutics.^[^ ^58^ ^]^ Notably, many bioactive molecules can autonomously undergo liquid–liquid phase separation and form more stable coacervate vesicles,^[^ ^23^ ^]^ opening the door to self‐contained carrier‐cargo systems.

## 4 Conclusion and Outlook

Coacervate vesicles represent a recent significant development of bottom‐up synthetic biology, combining the peculiar properties of coacervates, such as chemical simplicity, molecular enrichment, and enhanced reactivity, with the structural advantages of vesicular systems. A wide variety of coacervate vesicles can be synthesised through different formation strategies, which can be classified into three categories: irreversible, reversible, and nonequilibrium, each defined by its unique self‐assembly pathway and properties. These routes enable the creation of highly modular, biocompatible, and functionally tunable protocells.

Their chemical versatility and facile synthesis position coacervate vesicles as adaptable platforms for fundamental research and applications. Potential spans from origins‐of‐life models and compartmentalised reaction networks to drug delivery, synthetic cell communication, and adaptive materials. Crucially, their properties emerge directly from their formation routes, offering unprecedented control in designing responsive, scalable systems. As our understanding of coacervate vesicles deepens, their integration into more and more advanced chemical systems will be considered.

Developments in life‐like applications open the door to engineered protocell systems with capabilities for mutual recognition, inter‐vesicle communication, and selective material exchange, which are the pillars of synthetic life.^[^ ^59^ ^]^ Looking ahead, coacervate vesicles, as programmable, scalable, and biocompatible microcompartments, hold ever‐expanding potential for next‐generation soft‐matter technologies. In areas such as iontronics and soft robotics, these systems could prove particularly transformative by enabling the formation of functional tissue‐like architectures. This capability may pave the way for advanced bioinspired devices capable of sensing, actuation, and communication at the soft‐matter interface.^[^ ^60^, ^61^ ^]^ Finally, coacervate vesicles can be further engineered to serve as therapeutic platforms, ranging from gene delivery and enzymatic therapies to responsive drug depots, which may represent a powerful new direction in soft‐matter‐based medicine.^[^ ^62^ ^]^

## Conflict of Interest

The authors declare no conflict of interest.

## Data Availability

Data sharing is not applicable to this article as no new data were created or analysed in this study.
